# Emergency-physician Performed, Ultrasound-guided Lateral Femoral Cutaneous Nerve Block for Meralgia Paresthetica: A Report of Two Cases

**DOI:** 10.5811/cpcem.1409

**Published:** 2023-05-27

**Authors:** Matthew M. Kongkatong, Christopher D. Thom, Jakob Ottenhoff

**Affiliations:** University of Virginia Health System, Department of Emergency Medicine, Charlottesville, Virginia

**Keywords:** case series, meralgia paresthetica, regional anesthesia, lateral femoral cutaneous neve, ultrasound-guided nerve block

## Abstract

**Introduction:**

Neuropathy of the lateral femoral cutaneous nerve, also known as meralgia paresthetica, causes pain and paresthesia to the anterolateral thigh. It commonly results from nerve irritation from extrinsic compression; however, it may occur spontaneously. Symptoms from this condition can be debilitating, and the pain may be ascribed to other conditions leading to delays in diagnosis. Peripheral nerve blockade can be useful both diagnostically and therapeutically for meralgia paresthetica.

**Case Report:**

Two female patients in their sixties presented to the emergency department for chronic, atraumatic, left upper thigh pain. In both cases the patients had hyperalgesia and paresthesia to the anterolateral, upper thigh. The emergency physician performed an ultrasound-guided nerve block of the lateral femoral cutaneous nerve for each patient, which resulted in temporary, complete resolution of their pain.

**Conclusion:**

Meralgia paresthetica is an uncommon but painful condition that can elude diagnosis. Physical exam findings such as allodynia and hyperalgesia of the anterolateral thigh in the absence of back pain is suggestive of the diagnosis. Utrasound-guided nerve blockade can be helpful to the emergency physician to confirm the diagnosis and provide non-opioid pain relief to the patient.

## INTRODUCTION

Meralgia paresthetica (MP) also referred to as lateral femoral cutaneous nerve (LFCN) neuropathy is characterized by painful paresthesia and numbness of the anterolateral thigh. The incidence of this disorder is 4.3 per 10,000 person per year and usually presents in the third or fourth decade of life but can occur at any age. Causes of MP are grouped into spontaneous and iatrogenic categories. In spontaneous cases, external compression from tight clothing or belts can irritate the nerve. Compression may also result from intra-abdominal or pelvic processes, such as obesity, pregnancy, and uterine tumors. Spontaneous neuropathy can be caused by metabolic abnormalities such as diabetes, hypothyroidism, and nutritional deficiencies.^1^ Iatrogenic MP may result from inadvertent nerve transection from surgical incision, compression or traction from retractor placement, and extrinsic compression in the setting of body positioning for surgical exposure or restraint belt placement.^1^,^2^

Typically, patients will present with unilateral symptoms, but bilateral symptoms occur in 20% of patients.^2^ Patients often complain of uncomfortable tingling in the anterolateral thigh and can experience allodynia. Symptoms are often exacerbated by direct palpation of the area around the anterior superior iliac spine (ASIS) and by hip extension maneuvers.^1^,^2^ The presence of concurrent abdominal or urogenital complaints or abnormal examination findings like palpable masses should prompt the clinician to evaluate for other causes of the patient’s symptoms, which may involve abdominopelvic imaging such as ultrasound or computed tomography (CT).

Lumbar radiculopathy may mimic symptoms of MP, but in cases of isolated MP, back pain is absent. Muscle strength and deep tendon reflexes should be normal as the LCFN is a purely sensory nerve. Patients may have an antalgic gait.^1^,^2^ Symptoms and exam findings concerning for nerve root or spinal cord compression should be worked up with imaging, typically magnetic resonance imaging (MRI) or CT.

Meralgia paresthetica can cause significant patient distress and disability. Clinician unfamiliarity with this condition may lead to delay in diagnosis, leading to prolonged patient discomfort and unnecessary workup for other conditions.^2^ Diagnostic workup into the underlying cause depends on clinician suspicion for a secondary cause, such as compression from an abdominal mass.^1^,^2^ Nerve conduction studies may be performed as an outpatient but are usually not available to clinicians in the emergency department (ED). Diagnostic nerve blockade may be pursued in the ED or clinic. Rapid improvement or resolution of the patient’s symptoms after LFCN blockade can confirm the diagnosis as well as treat pain.^1^ Herein we present two cases where a patient presented to our ED with MP and received LFCN blocks.

## CASE REPORT

### Case 1

A 67-year-old female presented to the ED for left thigh pain worsening over the course of one month. The pain began after abdominal aortic aneurysm surgery three months prior. She had one ED presentation for severe pain as well as several clinic visits with workup including CT angiogram of the abdominal aorta with iliofemoral run-off and Doppler ultrasound for venous thrombosis without any surgical complications or venous thrombosis noted. Her vital signs on presentation were blood pressure (BP) of 183/88 millimeters of mercury (mm Hg), temperature 36.4°Celsius (C), heart rate 67 beats per minute (bpm), respiratory rate 20 breaths per minute, and oxygen saturation (SPO_2_) 98%. On physical examination, she had hyperalgesia and allodynia of the skin of her proximal, left anterolateral thigh. Tenderness and paresthesia to palpation inferior to the ASIS was present. She had normal strength to hip flexion, knee extension, and ankle dorsiflexion/plantarflexion and palpable ankle pulses.

Using a 6–15 megahertz linear array X-Porte (FUJIFILM Sonosite, Inc, Bothell, WA), we performed an ultrasound-guided LFCN block on the patient with seven milliliters (mL) of 0.25% bupivacaine after obtaining consent. On re-evaluation 10 minutes later, the patient reported complete resolution of the pain. She was given the diagnosis of MP and recommended to see pain management. On chart review follow-up, the initial block gave her two days of symptom relief. She received another block with pain management that lasted two days. She was started on pregabalin with improvement of symptoms over the next several weeks.

*CPC-EM Capsule*

What do we already know about this clinical entity?

*Meralgia paresthetica (MP) is an uncommon cause of upper thigh pain. While not life-threatening, it can be distressing for patients and difficult to diagnose*.

What makes this presentation of disease reportable?

*Because it is uncommon, pain from MP may be ascribed to other conditions leading to negative workups and repeat patient presentations for pain*.

What is the major learning point?

*Ultrasound-guided lateral femoral cutaneous nerve blockade is a low-risk option that can aid physicians in correctly diagnosing MP and provide non-opioid pain control*.

How might this improve emergency medicine practice?

*Correct diagnosis of MP in the emergency department allows physicians to initiate specific therapy and plan appropriate outpatient follow-up for MP patients*.

### Case 2

A 60-year-old female presented to the ED for acute worsening of a five-month history of atraumatic left thigh pain. The patient had multiple ED, primary care, and specialty care visits for the pain without definitive diagnosis. She had been worked up previously with negative Doppler ultrasound for venous thrombosis and MRI of the lumbar spine showing only mild neuroforaminal stenosis. On presentation she had normal vital signs: temperature 36.7°C, BP 128/73 mm Hg, respiratory rate 18 breaths per minute, and SPO_2_ 100% (besides mildly elevated heart rate of 99 bpm). On physical examination she had an antalgic gait, but a negative, straight-leg raise test bilaterally and normal strength to hip flexion, knee extension, and ankle movement bilaterally. She had normal ankle pulses. Skin examination was notable for hyperesthesia and allodynia to the region of the left, anterolateral thigh without erythema, induration, or rashes.

After consenting the patient, we performed an ultrasound-guided LFCN block with the X-Porte using six mL of 0.25% bupivacaine with four milligrams of preservative-free dexamethasone. After 10 minutes, the patient was pain-free and ambulated out of the ED with a normal gait. She was recommended to continue following up with her primary care physician; however, because they were outside our health system we could not perform chart follow-up.

## DISCUSSION

**Figure 1:**
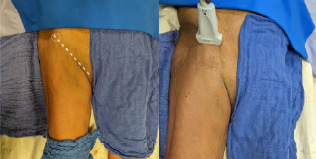
A) Anatomic drawing of the course of the lumbar plexus and lateral cutaneous femoral nerve (highlighted in green); B) dermatomal distribution of the lateral femoral cutaneous nerve (black arrow). Image obtained from https://commons.wikimedia.org/wiki/File:Gray824.png.

The LFCN is a purely sensory nerve originating from the posterior divisions of L2 and L3 of the lumbar plexus. The nerve courses in the retroperitoneal space through the psoas muscle and over the iliacus muscle in the region of the inguinal ligament (Figure 1).3 The most common (87%) anatomical position of the LFCN is deep to the inguinal ligament medial to the ASIS and sartorius muscle. However, there are variants described where the nerve may course through or superficial to the inguinal ligament, superficial or lateral to the ASIS, and through the sartorius muscle. Typically (79%), the nerve exits from the fascia lata and then divides into an anterior and posterior branch.^4^

**Image 1:**
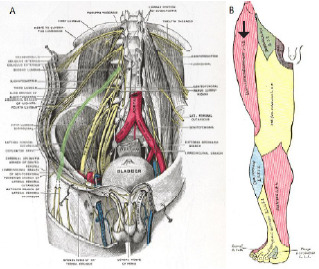
Superficial landmarks of the anterior superior iliac spine (ASIS) (circle) and inguinal ligament (dotted line). The transducer is placed axially just inferior to the ASIS to begin looking for the lateral femoral cutaneous nerve.

**Image 2:**
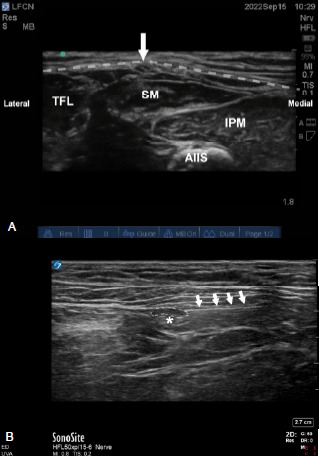
A) A labeled image of the lateral femoral cutaneous nerve (white arrow) lying deep to the fascia lata (dotted line) between the sartorius muscle (SM) and tensor fascia lata muscle (TFL). Also visualized is the illiopsoas muscle (IPM) medially and anterior inferior iliac spine (AIIS) in the far field; B) Image of a needle (white arrows) depositing local anesthetic (dotted area) around the lateral femoral cutaneous nerve (asterisk).

To perform the block, expose the affected thigh and inguinal area with the patient in a supine position. A high frequency linear transducer should be used to visualize the nerve and surrounding structures. The probe can initially be placed just inferior to the ASIS in a transverse orientation (Image 1). Scanning inferiorly, the nerve may be visualized as a round, hyperechoic, honeycombed structure deep to the subcutaneous tissue and fascia lata, positioned between the sartorius muscle medially and the tensor fascia lata muscle laterally (Image 2). As with other nerves, the LFCN may appear hypoechoic if the angle of insonation is oblique with respect to the nerve. This can be remedied by fanning the probe such that the nerve becomes hyperechoic on the screen.^3^,^5^ In cases of entrapment, the nerve may be a larger diameter than the unaffected side or have a more hypoechoic appearance of the fascicles. The ultrasound transducer can also be used to provoke a Tinel sign (elicitation of symptoms with palpation of the nerve) similar to sonographic Murphy’s sign in cholecystitis.^3^

Once the nerve is identified, the patient should be prepped in an antiseptic fashion. A probe cover and sterile conduction medium should be used. A small gauge needle is then guided to the soft tissue surrounding this nerve, and local anesthetic is deposited after negative aspiration of blood (Image 2). An in-plane or out-of-plane approach may be used; however, we prefer the in-plane approach to visualize the whole course of the needle.^5^ We prefer using a longer acting local anesthetic such as bupivacaine to maximize duration of effect; however, shorter acting local anesthetics such as lidocaine have been described. Volumes used in the literature range from 1 mL-10 mL and adjunctive use of steroids such as methylprednisolone and triamcinolone have been described.^6^–^10^ The use of adjunctive steroids has been shown to extend the duration of nerve blocks.^11^

Meralgia paresthetica is an uncommon entity that can cause significant discomfort. Conservative treatment of MP consists of addressing any extrinsic causes of compression like tight clothing and weight loss and use of non-steroidal anti-inflammatory drugs (NSAID). Persistent neuropathic pain may be treated with tricyclic antidepressants or anticonvulsants such as gabapentin.^2^ Nerve blockade of the LFCN offers a low-risk, non-opioid treatment for this condition.^1^ Although ultrasound-guided LFCN blockade has been described in the physical medicine and rehabilitation literature as well as the anesthesia literature; however, it has not commonly been described in the ED context. In both cases, the patients had severe pain that was refractory to treatment with acetaminophen, NSAIDs, and oxycodone. Both patients experienced rapid improvement after nerve blockade without any complications. In the first case, our diagnosis led the patient to be started on specific therapy for MP as opposed to renewed opioid prescription.

In both cases, the patients had undergone multiple comprehensive imaging studies and specialist evaluation without a diagnosis or improvement in symptoms. Performance of the LFCN block in the ED was diagnostic and therapeutic without any additional imaging studies or specialty consultation. In both cases our patients experienced great satisfaction with the result of the block. The first patient was able to be transitioned off opioid therapy for symptom control.

Limited studies of ultrasound-guided nerve blocks in MP patients show that some require only a single injection to improve symptoms. Tagliafico et al. showed symptom improvement in 16 out of 20 MP patients with a single injection. They also demonstrated a significant mean improvement of patient-reported quality of life.^6^ Another study performed by Klauser et al. showed that some patients may experience sustained pain control at 12 months after a single injection. However, most patients (15/20) required more than one block to be pain-free at 12 months.^9^ This demonstrates the importance of outpatient follow-up for MP that is diagnosed in the ED.

Ultrasound-guided nerve blocks have been shown to be efficacious for the treatment of musculoskeletal pain and neuropathic pain.^12^,^13^ With the progressive integration of ultrasound in emergency medicine practice, ultrasound-guided nerve blockade for procedural analgesia, fracture analgesia, and atraumatic conditions such as sciatica, has become more commonplace.^14^,^15^ The LFCN is a superficial, purely sensory nerve that is amenable to visualization on ultrasound. Additionally, the nerve is typically not directly adjacent to significant neurovascular structures, such as the femoral vasculature, making it a low-risk target amenable to even the novice ultrasound operator. Finally, only a small volume of anesthetic is needed to achieve the desired effect, decreasing the risk of local anesthetic toxicity. This makes it an attractive block for the emergency clinician to diagnose and treat MP. Our report adds to the body of literature demonstrating that ultrasound-guided peripheral nerve blockade can be effectively and safely performed in the ED.

## CONCLUSION

Meralgia paresthetica, although uncommon, can be a painful and chronic condition that may go misdiagnosed. Ultrasound-guided peripheral nerve blockade performed in the ED can help the emergency physician differentiate MP from other entities, which can improve diagnostic confidence and avoid potentially unnecessary workups. Ultrasound-guided LFCN blockade also provides the emergency physician an opioid-sparing option for treatment of MP in ED patients.

## Supplementary Information
